# Mood Symptoms are Associated With Cognitive Status, Brain Amyloid-Beta Deposition, and Plasma Biomarkers

**DOI:** 10.1155/da/7515712

**Published:** 2025-11-14

**Authors:** Jingru Wang, Lin Huang, Linfang Sun, Qihao Guo, Yingzi He, Yanping Jing

**Affiliations:** ^1^Department of Gerontology, Shanghai Sixth People's Hospital Affiliated to Shanghai Jiao Tong University School of Medicine, Shanghai 200233, China; ^2^Department of Nursing, Shanghai Sixth People's Hospital Affiliated to Shanghai Jiao Tong University School of Medicine, Shanghai 200233, China

**Keywords:** Alzheimer's disease, amyloid, mood symptoms, plasma biomarkers

## Abstract

**Background:**

Previous studies have indicated an association between mood symptoms and cognitive decline in the Alzheimer's disease (AD) spectrum. Amyloid-beta (Aβ) deposition in the brain, which is a core pathological characteristic of AD, along with the presence of plasma biomarkers, such as phosphorylated tau protein (p-tau), constitutes an early predictive indicator for AD. We attempted to explore the relationship between mood symptoms and the presence of AD-related plasma biomarkers in patients with brain Aβ deposition.

**Method:**

We included 2612 participants aged ≥50 years (707 males; average age 66.98 ± 7.75 years) in this study. We used the Hamilton depression rating scale (HAMD) and Hamilton anxiety rating scale (HAMA) to assess mood symptoms. Cognitive status was categorized into AD, mild cognitive impairment (MCI), subjective cognitive decline (SCD), and normal cognition (NC). We used analysis of covariance (ANCOVA) to compare mood symptoms assessment scores in different cognitive groups after making adjustments for age, gender, and education. Linear regression analysis was used to investigate the association between mood scores and plasma biomarker levels, adjusting for positivity in Aβ PET imaging.

**Results:**

Compared to NC patients, patients with AD exhibited higher levels of depression (mean of 4.72 versus 3.39, *p* < 0.05), whereas patients with SCD exhibited higher levels of anxiety (mean of 6.28 versus 4.26, *p* < 0.05). After accounting for brain Aβ deposition and presence of plasma biomarkers, the plasma neurofilament light chain (NFL) levels (*B* = 0.211, SE = 0.059, *p*=0.001) were associated with HAMD scores. The plasma p-tau181 levels (*B* = 1.328, SE = 0.576, *p*=0.025) were associated with HAMA scores.

**Conclusion:**

Plasma biomarkers have significant potential in predicting anxiety and depressive symptoms in individuals with brain Aβ deposition. This can aid the early clinical diagnosis and intervention of AD.

## 1. Introduction

The primary histological characteristics of Alzheimer's disease (AD) are the presence of amyloid-beta (Aβ) senile plaques and neurofibrillary tangles formed by hyperphosphorylated tau-protein. Clinically, these symptoms manifest as progressive cognitive decline, indicating underlying synaptic loss and neurodegeneration [1]. In addition to cognitive impairment, mood symptoms, such as depression and anxiety are important clinical manifestations in AD and are closely associated with cognitive function decline [2]. Research consistently demonstrates that mood symptoms are commonly observed in patients with different stages of AD. A significant percentage of patients with subjective cognitive decline (SCD), mild cognitive impairment (MCI), and AD dementia experience at least one clinically significant mood symptom [3]. Among these, anxiety and depression persist throughout AD progression, even in individuals with normal cognition (NC), and they are significantly associated with brain Aβ deposition [4]. Notably, depression is particularly prevalent in patients with MCI, with a prevalence rate of 32% [5]. Patients with MCI with comorbid depression tend to experience more rapid cognitive decline than patients without depression [6]. Furthermore, for patients with SCD, anxiety was found to increase the risk of clinical progression to MCI or dementia more frequently than depression [7]. For such patients with AD, the frequency and severity of mood symptoms tend to increase alongside their worsening cognition [8, 9]. However, the specific mechanisms underlying the association between mood symptoms and AD are yet to be elucidated. Recent evidence suggests that damage to the frontal-limbic circuitry is associated with depression, whereas damage to the amygdala circuitry is associated with anxiety [10]. Mood symptoms were also proposed to result from pathological changes in AD, but there may be underlying mechanisms that are currently unknown. These findings indicate that mood symptoms not only occur as comorbid symptoms during the course of AD but also play a significant role in AD pathogenesis. Furthermore, mood symptoms can potentially serve as early markers for identifying and predicting AD.

In recent years, as per the National Institute on Aging and Alzheimer's Association (NIA-AA) research framework, researchers have developed various quantitative methods and tools for assessing AD-associated pathological changes in the brain [11]. These advancements offer new possibilities for further investigations into the mechanistic association between mood symptoms and AD. Among these methods, traditional AD biomarker detection primarily relies on cerebrospinal fluid (CSF) sampling and positron emission tomography (PET) imaging techniques to accurately detect AD-related pathological changes in the brain. Currently, extensive research is being conducted on the association between mood symptoms, such as depression and brain Aβ deposition. However, the findings reported are inconsistent. Some cross-sectional studies have indicated that depression and anxiety symptoms are associated with higher levels of Aβ deposition, whereas some longitudinal studies have reported a link among higher levels of depression and anxiety, increased brain Aβ deposition, and cognitive decline [4]. A study has suggested that Aβ deposition, along with anxiety, but not depression, increases the risk of developing MCI [12]. Similar large-scale studies are needed to validate these findings. Furthermore, compared to traditional methods, plasma-based AD biomarkers offer advantages, such as less invasiveness, greater cost-effectiveness, and greater feasibility of use in a clinical setting [13]. The most extensively studied plasma biomarkers for AD pathology include Aβ42, Aβ40, phosphorylated tau (p-tau), total-tau (t-tau), and neurofilament light chain (NFL). Among these, NFL and plasma p-tau show promise as markers for AD [14, 15]. The latest research findings have highlighted the role of plasma NFL in neuropsychiatric symptoms (NPSs) across the AD continuum, demonstrating that elevated NFL levels are associated with an increased risk of psychosis in patients with brain Aβ deposition, as measured using the Neuropsychiatric Inventory Questionnaire (NPI-Q) [16]. Notably, evidence demonstrates that plasma Aβ42 and p-tau isoforms (p-tau 181, p-tau 217, and p-tau 231) are recognized as Core 1 biomarkers in the revised criteria, as the CSF counterpart, and amyloid PET [17]. However, research on the relationship between AD-related plasma biomarkers and mood symptoms remains limited and primarily focused on the association between depression and the presence of plasma Aβ42 or Aβ42/Aβ40 [18, 19]. Therefore, further research is needed to systematically explore the relationship between AD and mood symptoms by assessing the presence of plasma biomarkers (such as NFL and p-tau) and brain Aβ deposition.

In this study, we first examined the association between mood symptoms and various cognitive statuses. Following this, for patients with brain Aβ deposition, we explored the relationship between mood symptoms and AD-related plasma biomarkers. By doing so, we attempted to better understand the connection between mood symptoms and AD, providing valuable insights into the early diagnosis and prediction of preclinical AD as well as for guiding personalized treatment and interventions.

## 2. Methods

### 2.1. Study Design and Setting

**Figure 1:**
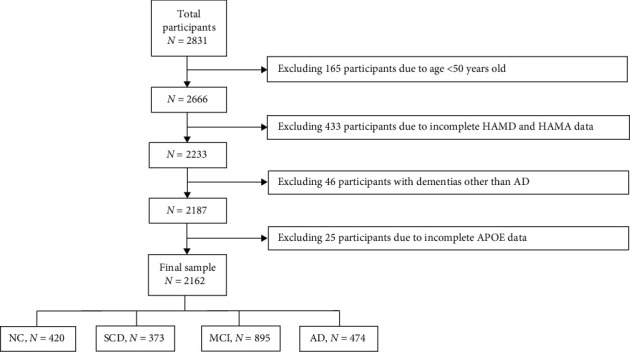
Flowchart of subjects enrolled.

This study is a cross-sectional analysis utilizing data from participants enrolled in the China Preclinical AD Study (C-PAS) cohort. Detailed recruitment methods, inclusion and exclusion criteria, data collection procedures, and ethical considerations are described in our previous publication [20]. Ultimately, data from 2162 individuals aged 50 years or older were included and analyzed (66.98 ± 7.75 years; 707 males) (Figure 1). This study was approved by the Ethics Committee of Shanghai Sixth People's Hospital (Approval ID: 2019-032) and complied with the Declaration of Helsinki. Written informed consent was obtained from each participant.

### 2.2. Clinical Diagnosis

Participants underwent comprehensive evaluations, including history of disease, neuropsychological testing, and examinations. Cognitive function was assessed using the Montreal cognitive assessment-basic (MoCA-B) [21] and Addenbrooke's cognitive examination III (ACE-III) [22]. AD with dementia was defined according to the NIA-AA 2011 criteria for probable AD [23]. MCI was defined by an actuarial neuropsychological method proposed by Edmonds et al. [24]. SCD included individuals with self-perceived cognitive decline and related concerns, based on SCD-plus guidelines of Jessen et al. [25]. NC were individuals with no memory concerns and no objective cognitive impairments, screened according to SCD criteria.

### 2.3. Mood Symptoms

We assessed mood symptoms using the Hamilton depression rating scale (HAMD) [26], the Hamilton anxiety rating scale (HAMA) [27], and DSM-IV criteria. For our analyze, HAMD (17 items) scores ≥8 indicated depression [28], and HAMA (14 items) scores ≥7 indicated anxiety [29]. Then participants were categorized into three groups: depression, anxiety, and depression with anxiety. HAMD and HAMA scores were also used as continuous variables, with higher scores indicating more severe symptoms. For dataset harmonization, mood symptoms were dichotomized as present (depression and/or anxiety) or absent (no depression or anxiety).

### 2.4. PET Data Acquisition

The participants underwent Aβ PET imaging with [18^F^]-AV45 (commercial name: amyvid, florbetapir) on PET/CT scanner (Biograph 64PET/CT, Siemens, Erlangen, Germany) at the Huashan Hospital, Fudan University (Shanghai, China), the methodology has been described in detail previously [20]. Then the normal and abnormal amyloid-PET scans (dichotomization into amyloid-positive/negative groups) were determined according to published criteria [30]. PET images were independently distinguished by three radiologists and the final diagnosis was reached by agreement between at least two radiologists.

### 2.5. Plasma-Derived AD Biomarkers

Participant's plasma is collected in memory clinic after an overnight fast, and centrifuged, aliquoted, and stored at −80°C. The concentrations of plasma Aβ42, Aβ40, t-tau, tau phosphorylated at threonine 181 (p-tau 181), and NFL were determined by single molecule array (Simoa) technology. Previous study has reported whole details [20]. In this study, the plasma-derived AD biomarkers were analyzed as continuous variables.

### 2.6. Statistical Analysis

All statistical analyses were performed using the SPSS 25.0 edition. The descriptive characteristics for categorical variables were summarized as percentages, and significant differences were evaluated using a *χ*^2^ test. Continuous variables were summarized as mean ± standard deviation (SD), and comparisons were performed using the *t*-test. Then, we explore the association between mood symptoms assessment scores and various cognitive status by analysis of covariance (ANCOVA) and adjusted age, gender, and education. Bonferroni-corrected post hoc tests were then applied for group-wise comparisons. Partial correlation analysis was conducted to evaluate the association between mood assessment scores (HAMA and HAMD) and cognitive scores (MoCA-B and ACE-III). Further, linear regression model was used to examine the association of mood symptoms assessment scores with plasma biomarkers levels in patients with brain Aβ deposition. We generated models adjusted for age, gender, APOE ε4 status, education, and history of disease. All of the tests were two-tailed, and the differences were considered to be statistically significant at *p* < 0.05.

## 3. Results

### 3.1. General Characteristics of the Participants

We included 2162 participants (707 males and 1455 females) with an average age of 66.98 ± 7.75 years who met the inclusion criteria for analysis (Figure 1). Of these, 420 participants were from the NC group, 373 participants from the SCD group, 895 participants from the MCI group, and 474 participants from the AD group.

The demographic characteristics and distribution of covariates in the different groups are presented in Table 1. The participants were categorized into four groups based on mood symptoms: without mood symptoms, depression, anxiety, and depression with anxiety group. Intergroup comparisons were conducted between the group without mood symptoms and each of the three other groups. We found that participants without mood symptoms tended to have higher mean scores on both MoCA-B (21.65 ± 5.84) and ACE-III (71.46 ± 15.62). Specifically, compared to that group, the depression group had significantly lower ACE-III scores (*p*=0.011), and the depression with anxiety group had significantly lower scores on both cognitive tests (both *p* < 0.05). Females constituted the majority in all groups, accounting for 62.90% of participants without mood symptoms, 71.90% of participants with anxiety, and 79.10% of participants with comorbid depression and anxiety. Both the anxiety group (*p*=0.002) and the depression with anxiety group (*p* < 0.001) had more female participants. Additionally, participants in these two groups had lower education years (*p*=0.030 and *p* < 0.001, respectively). The distribution of Aβ-PET (+) only exhibited statistically significant differences in the comparisons between the anxiety group and the group without mood symptoms (*p*=0.047). In contrast, the APOE status did not show any significant association with mood symptoms.

### 3.2. Association of Mood Symptoms With Cognitive Status

Table 2 shows the frequency of NC, SCD, MCI, and AD with mood symptoms present. The *χ*^2^ test results revealed significant differences in the distribution of mood symptoms in patients with different cognitive statuses (*χ*^2^ = 37.092, *p* < 0.001). Specifically, compared with participants in the NC group, more than one-third of participants in the SCD, MCI, and AD groups exhibited mood symptoms. With respect to the distribution of mood symptoms, depression with anxiety was most prevalent among the groups, followed by only anxiety and only depression. The highest proportion of depression was observed in the AD group (5.06%), whereas the highest proportion of anxiety was observed in the SCD group (19.84%).

To examine the variations in depression and anxiety scores among the different cognitive status groups, we performed an ANCOVA with adjustments for age, gender, and education (Table 2). We observed significant differences in depression and anxiety scores in groups with different cognitive statuses (both *p* < 0.001). Further pairwise comparisons revealed that the SCD, MCI, and AD groups had higher HAMD and HAMA scores than the NC group (all *p* < 0.05). Notably, the AD group exhibited the highest depression score (mean 4.72), whereas the SCD group exhibited the highest anxiety score (mean 6.28). In addition, the MoCA-B and ACE-III scores both demonstrated significant differences in individuals with different cognitive statuses (both *p* < 0.001). Further pairwise comparisons revealed that no statistically significant difference was observed exclusively between the NC and SCD groups.

We next analyzed the relationship between cognitive and mood scores using partial correlation in Table 3. After adjustments were made for age, gender, and education, we observed that higher HAMD scores were associated with lower MoCA-B scores (*r* = −0.070, *p*=0.001) and lower ACE-III scores (*r* = −0.098, *p* < 0.001). Higher HAMA scores were also associated with lower ACE-III scores (*r* = −0.070, *p*=0.001) but exhibited no significant correlation with MoCA-B scores.

### 3.3. Association of Mood Symptoms With Plasma Biomarkers in Aβ-PET (+)

We investigated the association between mood symptom scores and plasma-derived AD biomarker levels in individuals with mood symptoms (NC = 7, SCD = 12, MCI = 26, and AD = 17) (Tables 4 and 5).

As shown in Table 4, a statistically significant association was observed between plasma NFL levels and HAMD scores (*B* = 0.202, SE = 0.059, *p*=0.001), which remained significant even after adjustments were made for age and gender (*B* = 0.209, SE = 0.058, *p*=0.001). Further adjustment for APOE status, education level, and history of disease revealed that the association between plasma NFL levels and HAMD scores remained statistically significant (*B* = 0.211, SE = 0.059, *p*=0.001).

In Table 5, we observed a statistically significant association between plasma p-tau 181 levels and HAMA scores (*B* = 1.231, SE = 0.592, *p*=0.042), which remained significant after adjustments were made for covariates (*B* = 1.328, SE = 0.576, *p*=0.025).

We also assessed the association between mood symptom scores and plasma-derived AD biomarker levels in the Aβ-PET negative group (Tables S1 and S2). None of these associations reached statistical significance.

## 4. Discussion

In this study, we explored the complex association between mood symptoms, primarily depression and anxiety, and AD by integrating data on brain Aβ deposition and plasma biomarkers. Our findings elucidate several key aspects of this association. First, our results supported the prevailing notion that depression and anxiety are prevalent among patients with varying cognitive statuses. Specifically, depression is more pronounced in patients with AD, whereas anxiety is particularly prominent in patients with SCD. We analyzed plasma biomarkers in patients with brain Aβ deposition. The results indicate a statistically significant association between HAMD and HAMA scores and plasma biomarker levels. Notably, among patients with mood symptoms, the HAMD score is associated with NFL levels, whereas the HAMA score is closely correlated with the p-tau 181 levels. These findings provide crucial insights into the intrinsic link between mood symptoms and AD, paving the way for related research in the future.

### 4.1. Association of Mood Symptoms With Cognitive Status

Our results indicate that participants with SCD, MCI, and AD have significantly higher levels of depression and anxiety than NC individuals. Higher mood symptom scores exhibit a significant negative correlation with poorer cognitive performance, confirming previous findings [3]. The elevated mood symptoms may be attributed to damage in the frontal-limbic and amygdala circuits [10]. The frontal-limbic circuit, which is closely linked to emotional regulation and self-esteem, can lead to depressive symptoms when impaired. The amygdala circuit, which plays a critical role in fear and emotional responses, can increase susceptibility to anxiety when dysfunctional. Further observation revealed that the severity of mood symptoms varies among individuals with different cognitive statuses. Specifically, patients with AD patients exhibited the highest levels of depression, whereas patients with SCD exhibited the highest levels of anxiety. This suggests a unique association between different cognitive statuses and distinct mood symptoms. A cross-sectional study conducted in Norway using the NPI-Q showed that depression was the most common NPS in patients with MCI, whereas apathy was most prevalent in patients with AD, followed by depression. The NPS severity increased with declining cognitive function [31]. However, in our study, we did not observe the expected level of depression in patients with MCI, possibly owing to differences in the measurement tools used. Notably, depression is the second most common symptom after apathy in AD [31], aligning with our findings and highlighting the unique psychological state of patients with AD. Approximately two-thirds of our participants were female. Given that the prevalence and severity of depressive symptoms are higher among female patients with AD than male patients [32], gender differences may have influenced our results. Additionally, evidence suggests that patients with SCD have specific deficits in handling uncertainty, which contributes to their anxiety [33]. While the association between emotional symptoms and different cognitive statuses has been explored in different studies, the results have been inconsistent [34, 35]. We hypothesize the existence of a unique, non-linear association between cognitive status and various mood symptoms. Further investigations can provide valuable insights into this complex relationship.

Notably, our results present an interesting phenomenon: individuals with anxiety symptoms were less frequently Aβ-PET (+) compared to individuals without mood symptoms. However, a recent study of 1440 participants revealed no significant difference in Aβ deposition between individuals with and without anxiety [12]. This difference may be attributed to the limited sample size of patients undergoing PET imaging in our study, which may not fully represent the set of Aβ-PET (+) individuals. This limitation will be addressed in future research by expanding the sample size of Aβ-PET scans. Additionally, in our study, participants with mood symptoms always exhibited a stronger willingness to undergo Aβ-PET scans than participants without mood symptoms [20]. These individuals were more inclined to understand their risk of developing AD and were relatively more receptive to the potential risk of PET scanning [20]. We hypothesized that their memory deficits are more likely associated with their anxiety symptoms rather than the AD pathology. Hence, this did not result in a higher proportion of Aβ-PET-positive status in participants.

### 4.2. Association of Mood Symptoms With Plasma Biomarkers in Aβ-PET (+) Individuals

In the field of neurodegenerative disease research, the study of plasma biomarkers has garnered significant attention in recent years [36]. NFL, a marker of axonal damage, can be detected in both CSF and plasma [37]. In patients with AD, NFL is often associated with neuronal degeneration and is one of the potential peripheral biomarkers for neurodegenerative diseases [38]. When studying patients with brain Aβ deposition, we observed a close correlation between the AD plasma biomarker levels and the mood symptoms of patients. Specifically, higher NFL levels were significantly correlated with higher HAMD scores among patients presenting mood symptoms, potentially indicating that axonal damage plays an important role in depression pathophysiology. Previous studies have shown that elevated plasma NFL levels are associated with an increased risk of clinically relevant depressive symptoms [39] and may serve as a biomarker for depression diagnosis [40]. On one hand, neuronal damage and inflammatory responses can affect the normal function of key brain regions involved in emotional regulation, such as the prefrontal cortex and amygdala, which can lead to mood symptoms, such as depression [41, 42]. On the other hand, Schuurmans et al. [43] proposed that axonal damage is a potential common mechanism between depression and neurodegenerative diseases. The close association between NFL levels and depressive symptoms observed in this study may suggest a complex interaction between neuronal axonal damage in the brain and the network of emotional regulation in depression.

Similarly, CSF p-tau 181 is a key component in the formation of neurofibrillary tangles in patients with AD. Plasma p-tau 181 exhibits a moderate correlation with CSF p-tau 181 and can directly reflect the tau phosphorylation state in the brain [44, 45]. In this study, the plasma p-tau 181 levels were positively correlated with HAMA scores, indicating a connection between tau pathology and mood symptoms. Although limited evidence is available on the link between tau pathology and NPS in preclinical AD, NPS has been shown to be associated with tau deposition in specific brain regions [46]. Furthermore, higher levels of t-tau and p-tau in CSF are associated with anxiety [47], possibly owing to compensatory anxiety behaviors arising from cognitive impairment [48]. Early-onset mental illnesses may also increase the risk of dementia through mechanisms such as chronically elevated cortisol levels or increased neuroinflammation, which exert neurotoxic effects on the brain and can lead to pathological changes in AD [49–51]. Related studies have shown that higher plasma tau levels are associated with heightened NPSs [52], with higher levels of plasma-derived p-tau 181 and p-tau 217 specifically linked to increased appetite changes, irritability, and disinhibition symptoms. Additionally, a cohort study has shown that elevated plasma p-tau 181 levels are closely correlated with the occurrence of psychiatric symptoms (delusions and hallucinations) in AD, emphasizing the potential use of plasma p-tau 181 as a biomarker for neuropsychiatric diseases in AD [53]. These findings provide valuable insights into the association between plasma tau proteins and mood symptoms. However, further research is needed to explore the intrinsic connection between plasma tau proteins and symptoms, such as anxiety.

## 5. Limitation

This cross-sectional study revealed correlations between mood and cognition. However, as mood symptoms may change with time and in individuals with different cognitive statuses, long-term follow-up is needed to fully understand the relationship between mood and cognition. Additionally, we were unable to establish a definitive causal relationship between mood symptoms and plasma biomarkers. In the future, we plan to conduct cohort studies and expand the sample size of Aβ PET scans. We also intend to use additional indicators, including clinical diagnosis, detailed neuropsychological test scores, and AD-related biomarkers, to determine the stage of disease progression. By doing so, we aim to verify and further explore the association between mood and cognition. Furthermore, the participants in this study were volunteers from the local community, and the sample may not be fully representative of the local population.

## 6. Conclusion

In summary, our findings revealed that depression and anxiety are prevalent in individuals with different cognitive statuses, with depression being more prominent in patients with AD and anxiety being more prominent in patients with SCD. This suggests unique associations between cognitive status and mood symptoms. Additionally, HAMD and HAMA scores significantly correlate with plasma biomarkers, specifically with NFL levels for depression and p-tau 181 levels for anxiety in patients with mood symptoms. Overall, our findings improved our understanding of the complex relationship between mood symptoms and AD and provided important references for the early diagnosis and prediction of preclinical AD.

## Figures and Tables

**Table 1:** Characteristics of participants and comparisons (*N* = 2162).

Variables	Total (*N* = 2162)	Without mood symptoms (*N* = 1373)	Depression (*N* = 81)	Anxiety (*N* = 320)	Depression with anxiety (*N* = 388)	*P*_*a*_	*P*_*b*_	*P*_*c*_
Age (years)	2162	66.89 ± 7.85	66.80 ± 7.42	66.99 ± 7.19	67.33 ± 7.90	0.923	0.833	0.330
MoCA-B score	2136	21.65 ± 5.84	20.52 ± 6.73	21.61 ± 5.76	20.82 ± 6.13	0.107	0.927	0.016
ACE-III score	2138	71.46 ± 15.62	66.78 ± 18.50	70.59 ± 14.92	67.86 ± 16.91	0.011	0.368	0.002
Education (years)	2156	11.58 ± 3.56	11.61 ± 3.50	11.09 ± 3.58	10.93 ± 3.61	0.939	0.030	<0.001
Gender (%)	—	—	—	—	—	0.360	0.002	<0.001
Males	707 (32.70)	510 (37.14)	26 (32.10)	90 (28.13)	81 (20.88)	—	—	—
Females	1455 (67.30)	863 (62.86)	55 (67.90)	230 (71.87)	307 (79.12)	—	—	—
Marriage (%)	—	—	—	—	—	0.026	0.376	<0.001
Single	26 (1.28)	16 (1.17)	1 (1.33)	4 (1.32)	5 (1.36)	—	—	—
Married	1770 (87.15)	1151 (89.43)	61 (81.33)	260 (86.09)	298 (81.20)	—	—	—
Divorced	70 (3.45)	31 (2.40)	6 (8.00)	11 (3.64)	22 (6.00)	—	—	—
Widowed	165 (8.12)	89 (6.92)	7 (9.33)	27 (8.94)	42 (11.44)	—	—	—
Aβ-PET (+) (%)	—	—	—	—	—	0.915	0.047	0.416
Yes	286 (38.75)	191 (40.99)	12 (40.00)	31 (30.39)	52 (37.14)	—	—	—
No	452 (61.25)	275 (59.01)	18 (60.00)	71 (69.61)	88 (62.86)	—	—	—
APOE ε4 carriers (%)	—	—	—	—	—	0.238	0.218	0.882
Yes	558 (25.81)	359 (26.15)	26 (32.10)	73 (22.81)	100 (25.77)	—	—	—
No	1604 (74.19)	1014 (73.85)	55 (67.90)	247 (77.19)	288 (74.23)	—	—	—

*Note:* Continuous variables are presented as mean ± SD and categorical variables as number (percentage). *P*_*a*_: without mood symptoms group versus depression group; *P*_*b*_: without mood symptoms group versus anxiety group; *P*_*c*_: without mood symptoms group versus depression with anxiety group.

Abbreviations: Aβ, Amyloid-beta protein; ACE-III, Addenbrooke's Cognitive Examination III; APOE, Apolipoprotein E genotype; MoCA-B, Montreal cognitive assessment-basic; PET, positron emission tomography.

**Table 2:** Comparison of mood and cognitive measures among NC, SCD, MCI, and AD groups (*N* = 2162).

Variables	NC	SCD	MCI	AD	*χ* ^2^/*F*	*p*-Value
	*N* = 420	*N* = 373	*N* = 895	*N* = 474		
Mood symptoms	—	—	—	—	37.092	<0.001
Without mood symptoms	310 (73.81)	208 (55.76)	560 (62.57)	295 (62.24)	—	—
Depression	12 (2.86)	15 (4.02)	30 (3.35)	24 (5.06)	—	—
Anxiety	42 (10.00)	74 (19.84)	144 (16.09)	60 (12.66)	—	—
Depression and anxiety	56 (13.33)	76 (20.38)	161 (17.99)	95 (20.04)	—	—
HAMD score	3.39 ± 0.23	4.55 ± 0.25^a^	4.20 ± 0.16^a^	4.72 ± 0.23^a^	6.770	<0.001
HAMA score	4.26 ± 0.28	6.28 ± 0.30^a^	5.53 ± 0.19^a^	5.64 ± 0.27^a^	9.397	<0.001
MoCA-B score	25.01 ± 0.17	24.62 ± 0.18	22.22 ± 0.12^a^	13.91 ± 0.17^a,b,c^	849.271	<0.001
ACE-III score	80.45 ± 0.44	79.70 ± 0.48	73.27 ± 0.30^a,b^	51.38 ± 0.44^a,b,c^	893.232	<0.001

*Note:* categorical variables were tested using *χ*^2^ test. Continuous variables were presented as mean ± SD, and comparisons were performed using the analysis of covariance (ANCOVA). *p*-Value represent the result of Bonferroni corrections after adjusting for age, gender, and education.

Abbreviations: ACE-III, Addenbrooke's cognitive examination III; AD, Alzheimer's disease; HAMA, Hamilton anxiety scale; HAMD, Hamilton depression rating scale; MCI, mild cognitive impairment; MoCA-B, Montreal cognitive assessment-basic; NC, normal cognition; SCD, subjective cognitive decline.

^a^Compared with NC group, *p* < 0.05.

^b^Compared with SCD group, *p* < 0.05.

^c^Compared with MCI group, *p* < 0.05.

**Table 3:** Partial correlation between the cognitive scores and mood scores (*N* = 2162).

Mood scores	Cognitive scores	Crude		Adjusted	
		*r*	*p*-Value	*r*	*p*-Value
HAMD score	MoCA-B score	−0.101	<0.001	−0.070	0.001
	ACE-III score	−0.135	<0.001	−0.098	<0.001
HAMA score	MoCA-B score	−0.054	0.012	−0.032	0.137
	ACE-III score	−0.096	<0.001	−0.070	0.001

*Note:* Adjusted *p*-value represent the result of Bonferroni corrections after adjusting for age, gender, and education.

Abbreviations: ACE-III, Addenbrooke's cognitive examination III; HAMA, Hamilton anxiety scale; HAMD, Hamilton depression rating scale; MoCA-B, Montreal cognitive assessment-basic.

**Table 4:** Multivariable linear regression analysis of plasma biomarkers and HAMD score in mood symptoms with Aβ-PET (+) (*N* = 62).

Models	Plasma biomarkers	*B*	SE	*R* ^2^	*p*-Value
Crude model	T-tau	0.883	0.651	0.030	0.180
	Aβ42	−0.185	0.227	0.011	0.417
	Aβ40	0.010	0.012	0.010	0.435
	Aβ42/Aβ40	−59.661	45.494	0.028	0.195
	NFL	0.202	0.059	0.164	0.001
	p-tau 181	0.568	0.487	0.022	0.248
Model 1	T-tau	0.789	0.661	0.065	0.237
	Aβ42	−0.184	0.227	0.053	0.420
	Aβ40	0.008	0.014	0.048	0.561
	Aβ42/Aβ40	−51.659	48.719	0.060	0.293
	NFL	0.209	0.058	0.216	0.001
	p-tau 181	0.537	0.489	0.061	0.277
Model 2	T-tau	0.852	0.671	0.084	0.209
	Aβ42	−0.160	0.236	0.066	0.502
	Aβ40	0.010	0.014	0.067	0.476
	Aβ42/Aβ40	−52.864	49.827	0.076	0.293
	NFL	0.211	0.059	0.233	0.001
	p-tau 181	0.611	0.506	0.082	0.233

*Note:* Model 1: age and gender. Model 2: age, gender, APOE e4 status, and education. p-tau 181, tau phosphorylated at threonine 181.

Abbreviations: Aβ: amyloid-beta protein; CI: confidence interval; HAMD, Hamilton depression rating scale; NFL, neurofilament light; PET, positron emission tomography; T-tau, total tau.

**Table 5:** Multivariable linear regression analysis of plasma biomarkers and HAMA score in mood symptoms with Aβ-PET (+) (*N* = 62).

Models	Plasma biomarkers	*B*	SE	*R* ^2^	*p*-Value
Crude model	T-tau	1.330	0.805	0.043	0.104
	Aβ42	0.013	0.284	0.000	0.963
	Aβ40	0.016	0.015	0.019	0.290
	Aβ42/Aβ40	−51.558	57.099	0.013	0.370
	NFL	0.122	0.079	0.039	0.126
	p-tau 181	1.231	0.592	0.067	0.042
Model 1	T-tau	1.078	0.772	0.177	0.168
	Aβ42	0.021	0.268	0.149	0.938
	Aβ40	0.011	0.016	0.156	0.493
	Aβ42/Aβ40	−20.734	57.695	0.151	0.721
	NFL	0.136	0.073	0.196	0.069
	p-tau 181	1.160	0.560	0.208	0.043
Model 2	T-tau	1.152	0.785	0.193	0.148
	Aβ42	−0.036	0.278	0.162	0.897
	Aβ40	0.010	0.017	0.167	0.564
	Aβ42/Aβ40	−29.880	59.013	0.165	0.615
	NFL	0.146	0.074	0.216	0.055
	p-tau 181	1.328	0.576	0.234	0.025

*Note:* Model 1: age and gender. Model 2: age, gender, APOE e4 status, and education. p-tau 181, tau phosphorylated at threonine 181.

Abbreviations: Aβ, amyloid-beta protein; CI, confidence interval; HAMA, Hamilton anxiety scale; NFL, neurofilament light; PET, positron emission tomography; T-tau, total tau.

## Data Availability

The data that support the findings of this study are available from the corresponding author.

## Supporting Information

Supporting Information Table S1. This table presents the multivariable linear regression analysis of plasma biomarkers and HAMD score in mood symptoms with Aβ-PET negative group. Table S2. This table presents the multivariable linear regression analysis of plasma biomarkers and HAMA score in mood symptoms with Aβ-PET negative group.
