# Supplementary material for: Mood Symptoms are Associated With Cognitive Status, Brain Amyloid-Beta Deposition, and Plasma Biomarkers
Source: Depress Anxiety. 2025 Nov 14;2025:7515712. doi: 10.1155/da/7515712 (PMC12638140; doi:10.1155/da/7515712)
Supplement: Supporting Information — Table S1. This table presents the multivariable linear regression analysis of plasma biomarkers and HAMD score in mood symptoms with Aβ-PET negative group. Table S2. This table presents the multivariable linear regression analysis of plasma biomarkers and HAMA score in mood symptoms with Aβ-PET negative group. [file 7515712.f1.docx]

**TABLE S1: Multivariable linear regression analysis of plasma biomarkers and HAMD score in mood symptoms with Aβ-PET (-). (N=123)**

| **Models** | **Plasma**  **biomarkers** | ***B*** | ***SE*** | ***R^2^*** | ***P*** |
| --- | --- | --- | --- | --- | --- |
| **Crude model** |  |  |  |  |  |
|  | T-tau | -0.127 | 0.345 | 0.001 | 0.714 |
|  | Aβ42 | 0.040 | 0.118 | 0.001 | 0.736 |
|  | Aβ40 | 0.003 | 0.007 | 0.001 | 0.708 |
|  | Aβ42/Aβ40 | -10.262 | 31.176 | 0.001 | 0.743 |
|  | NFL | 0.024 | 0.027 | 0.006 | 0.383 |
|  | P-tau181 | 0.088 | 0.398 | 0.000 | 0.826 |
| **Model 1** |  |  |  |  |  |
|  | T-tau | -0.119 | 0.349 | 0.003 | 0.734 |
|  | Aβ42 | 0.037 | 0.120 | 0.003 | 0.756 |
|  | Aβ40 | 0.002 | 0.007 | 0.003 | 0.813 |
|  | Aβ42/Aβ40 | -6.566 | 32.703 | 0.003 | 0.841 |
|  | NFL | 0.022 | 0.029 | 0.007 | 0.452 |
|  | P-tau181 | 0.064 | 0.412 | 0.003 | 0.877 |
| **Model 2** |  |  |  |  |  |
|  | T-tau | -0.103 | 0.357 | 0.016 | 0.772 |
|  | Aβ42 | 0.033 | 0.125 | 0.016 | 0.795 |
|  | Aβ40 | 0.002 | 0.007 | 0.016 | 0.768 |
|  | Aβ42/Aβ40 | -11.795 | 35.031 | 0.016 | 0.737 |
|  | NFL | 0.019 | 0.029 | 0.019 | 0.521 |
|  | P-tau181 | 0.093 | 0.418 | 0.016 | 0.824 |

**Note:** **Model 1**: age, gender; **Model 2**: age, gender, APOE ε4 status, education.

**Abbreviation:** T-tau: Total tau; Aβ: Amyloid-beta protein; PET: positron emission tomography; NFL: Neurofilament Light; P-tau181: Tau phosphorylated at threonine 181; CI: confidence interval; HAMD, Hamilton Depression Rating Scale.

**TABLE S2: Multivariable linear regression analysis of plasma biomarkers and HAMA score in mood symptoms with Aβ-PET (-). (N=123)**

| **Models** | **Plasma**  **biomarkers** | ***B*** | ***SE*** | ***R^2^*** | ***P*** |
| --- | --- | --- | --- | --- | --- |
| **Crude model** |  |  |  |  |  |
|  | T-tau | -0.154 | 0.382 | 0.001 | 0.688 |
|  | Aβ42 | -0.012 | 0.131 | 0.000 | 0.930 |
|  | Aβ40 | -0.011 | 0.008 | 0.018 | 0.135 |
|  | Aβ42/Aβ40 | 47.186 | 34.232 | 0.015 | 0.171 |
|  | NFL | 0.031 | 0.030 | 0.009 | 0.306 |
|  | P-tau181 | -0.036 | 0.440 | 0.000 | 0.936 |
| **Model 1** |  |  |  |  |  |
|  | T-tau | -0.130 | 0.383 | 0.018 | 0.735 |
|  | Aβ42 | -0.029 | 0.132 | 0.017 | 0.826 |
|  | Aβ40 | -0.013 | 0.008 | 0.038 | 0.107 |
|  | Aβ42/Aβ40 | 45.816 | 35.672 | 0.030 | 0.202 |
|  | NFL | 0.032 | 0.031 | 0.026 | 0.302 |
|  | P-tau181 | 0.087 | 0.453 | 0.017 | 0.848 |
| **Model 2** |  |  |  |  |  |
|  | T-tau | -0.160 | 0.389 | 0.032 | 0.682 |
|  | Aβ42 | -0.083 | 0.136 | 0.034 | 0.542 |
|  | Aβ40 | -0.012 | 0.008 | 0.051 | 0.121 |
|  | Aβ42/Aβ40 | 30.273 | 38.141 | 0.036 | 0.429 |
|  | NFL | 0.026 | 0.032 | 0.037 | 0.407 |
|  | P-tau181 | -0.108 | 0.457 | 0.031 | 0.813 |

**Note:** **Model 1**: age, gender; **Model 2**: age, gender, APOE ε4 status, education.

**Abbreviation:** T-tau: Total tau; Aβ: Amyloid-beta protein; PET: positron emission tomography; NFL: Neurofilament Light; P-tau181: Tau phosphorylated at threonine 181; CI: confidence interval; HAMA, Hamilton Anxiety Scale.
